# A model for habitus-adjusted paediatric weight estimation by age and data concerning the validation of this method on a large dataset of English children

**DOI:** 10.1016/j.dib.2017.11.094

**Published:** 2017-12-06

**Authors:** Nicholas Appelbaum, Jonathan Clarke, Ian Maconochie, Ara Darzi

**Affiliations:** a Department of Surgery and Cancer, Division of Surgery, Imperial College London, United Kingdom; b Department of Emergency Medicine, Division of Medicine, Imperial College London, United Kingdom

## Abstract

It is often not possible to weigh children upon arrival at an emergency room before commencing the provision of emergency care. Because drugs for children are prescribed on the basis of age and body weight, estimations of weight are necessitated. Age-based equations have been one of the most commonly used weight estimation strategies historically. Due to the variability of weight for age in children, and variations in body habitus, these methods are inaccurate by design (Young and Korotzer, 2016) [1].

**Specifications Table**

**Table:** 

Subject area	*Resuscitation Science*
More specific subject area	*Paediatric weight estimation*
Type of data	*Table, graphs*
How data was acquired	*Performance data were acquired by computer based analysis of data from the 2015/2016 year of the UK National Child Measurement Program. Model data were derived from CDC centile data.*
Data format	*Analysed performance data with exported model*
Experimental factors	*Pretreatment of NCMP data for experimental aspects:*
	*Data was suppressed by NHS Digital in line with the NHS Anonymisation Standard. Extreme outliers of age-for-weight above the 99.995th percentile (3192 records) and below the 0.005th percentile (631 records) were removed from the dataset. In addition to this, 89,260 records were suppressed where the local authority code and a locally small population might have allowed for identification of an individual.*
	*To map the NCMP data onto the CDC data, child age-in-months was rounded to the nearest half-month. Limited secondary data cleaning, removing extreme outliers for age was performed: in reception year, age in months 48.5, 49.5, and 70.5, and for Year 6 children, 120.5, 141.5, and 142.5 months.*
Experimental features	*Data concerning the performance of the Helix Weight Estimation Tool for 1,076,743 English children aged 4–5 and 10–11 years.*
Data source location	*The NCMP collects data from all children in the UK in Reception Year and Year 6 of their school careers, in all local authorities in the UK.*
Data accessibility	*Both NCMP data and CDC data are made freely available*
	*NCMP:*https://digital.nhs.uk/catalogue/PUB22269[2]*CDC:*https://www.cdc.gov/growthcharts/percentile_data_files.htm[3]
Related research article	*Paediatric weight estimation by age in the digital era: optimising a necessary evil (Resuscitation, in press)*

**Value of the Data**

• Provide a model for assessment of this method in other populations
• Aid researchers in understanding the extent to which age-based weight estimation strategies may be improved by taking into account a habitus assessment
• Serve as a methodological and performance benchmark for further attempts to optimise paediatric emergency weight estimation by age.

## 1 Data

• A Bland Altman plot of the performance of the Helix Method on the NCMP dataset.
• An exported model of the Helix method which may be used by other research groups to interrogate this technique in different populations.

## 2 Experimental design, materials, and methods

As described in the original paper (*Resuscitation, in press*), the NCMP and CDC data were processed using the Python programming language (Python Software Foundation. Python Language Reference, version 3.6), and the *pandas* library (version 0.20.2, http://pandas.pydata.org), in the Jupyter Notebook computational environment (https://jupyter.org).

The Helix method uses 7 habitus scores, each mapping closely onto the habitus scores used by Wells et al. in the PAWPER-XL system [4]. We assigned CDC weight-for-age and BMI-for-age centiles to each habitus score as follows: HS1=10th, HS2=25th, HS3=50th, HS4=75th, HS5=90th, HS6=95th, and HS=97th centiles. Each child in the dataset was allocated to the CDC BMI-for-age centile (10th, 25th, 50th, 75th, 90th, 95th, 97th) closest to their actual BMI from the NCMP dataset, and the corresponding CDC weight-for-age centile was used to determine an estimated weight.

The overall accuracy of the model was tested by calculating the mean percentage error as a measure of estimation bias. 95% limits of agreement were calculated as a measure of precision. Statistical analysis was performed using Stata (Stata-Corp. 2015. Stata Statistical Software: Release 14. College Station, TX: StataCorp LP). Detailed understanding of the performance of this model may be gained by reference to the original publication.

The table attached in the repository folder shows the estimated weights for an individual of a given age in months and gender, according to each of the seven habitus scores developed in our model, and which was used in the validation paper (*Resuscitation, in press*). The BMI values that pertain to the lower and upper limits of the habitus score are included for reference as Table 1. The estimates from the new APLS and EPLS formulae for given ages are also included for reference, accompanying the model description.

**Table 1:** BMI values pertaining to upper and lower limits of habitus scores.

Habitus category	Lower limit of BMI	Upper limit of BMI	Weight centile
1	N/A	B[3–25]	P[10]
2	B[3–25]	B[25–50]	P[25]
3	B[25–50]	B[50–75]	P[50]
4	B[50–75]	B[75–90]	P[75]
5	B[75–90]	B[90–95]	P[90]
6	B[90–95]	B[95–97]	P[95]
7	B[95–97]	N/A	P[97]

B[x-y] refers to the average of the two age and sex-specific BMI values for centiles x and y derived from CDC BMI for age centiles.

P[x] refers to the weight of centile x from CDC weight for age centiles.

**Fig. 1:**
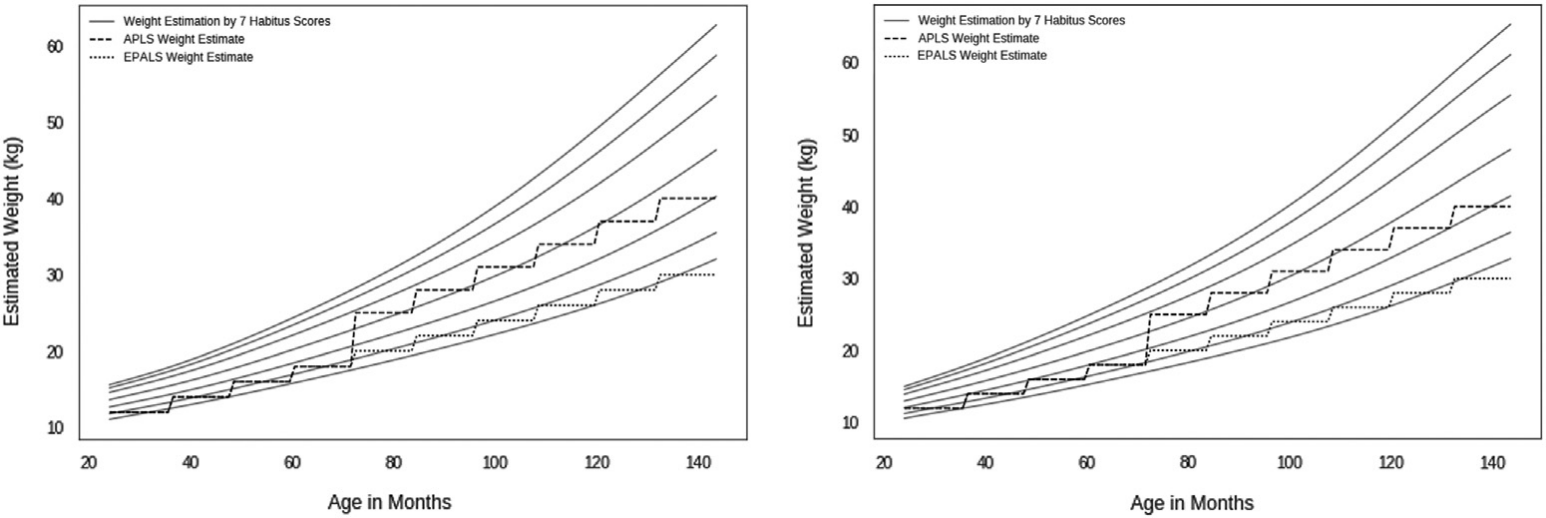
APLS and EPALS weight estimates compared to estimate spread with 7 habitus scores.

**Fig. 2:**
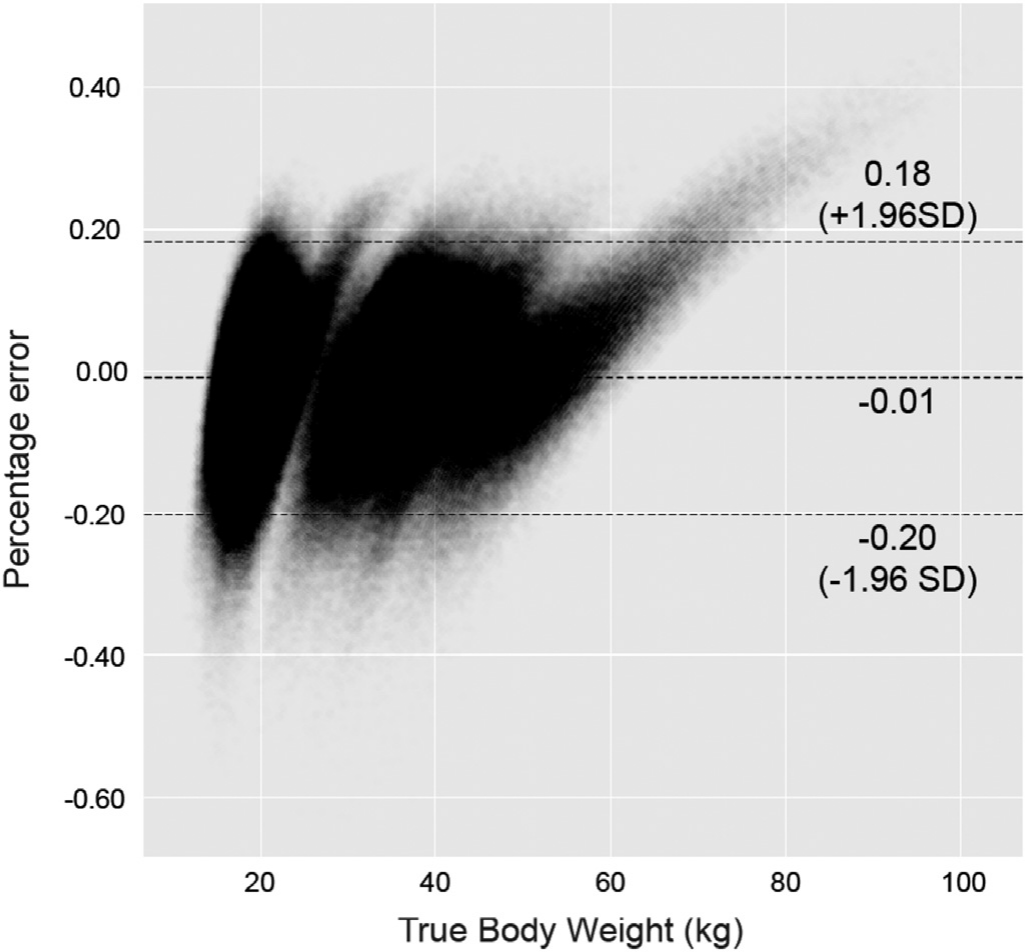
Bland Altman plot for overall samples. Modified Bland Altman plot for overall sample, with the percentage error presented as a fraction.

The two graphs (Fig. 1) demonstrate the spread of the estimated weights for each of the seven habitus scores between the ages of 24 months and 144 months in dark grey. Superimposed are the weight estimates according to the APLS and EPALS formulae. The graphs demonstrate that in both boys and girls, the current widely used formula based strategies significantly underestimate the weight of older children and fail to account for the broadening of the weight distribution with age. These charts make no assumptions about accuracy, but show how they compare numerically. Fig. 2 shows a Bland Altman plot for the Helix method.
